# Effect of Statin Therapy on Mortality and Recurrence of Intracerebral Hemorrhage in Patients With Spontaneous Intracerebral Hemorrhage

**DOI:** 10.7759/cureus.31150

**Published:** 2022-11-06

**Authors:** Sidra Jamil, Saima Batool, Tanveer Ahamad Shaik, Urooba Shakil, Tafseer Zahra, Mohammad Munim Zahoor, Venkata Anirudh Chunchu, Neelum Ali

**Affiliations:** 1 Internal Medicine, California Institute of Behavioral Neurosciences & Psychology, Fairfield, USA; 2 Internal Medicine, Hameed Latif Hospital, Lahore, PAK; 3 Cardiovascular Medicine, University of Louisville School of Medicine, Louisville, USA; 4 Internal Medicine, University of Health Sciences, Lahore, PAK; 5 Internal Medicine, Ghurki Trust Teaching Hospital, Lahore, PAK; 6 Medicine, Avalon University School of Medicine, Willemstad, CUW

**Keywords:** meta-analysis, cholestrol, statin therapy, mortality, intracranial hemorrhage

## Abstract

Statins can play an essential role in the tertiary and primary prevention of cardiovascular events by reduction of cholesterol in a stroke patient. This meta-analysis aims to assess statin therapy's effect on mortality and recurrence of Intracranial Hemorrhage (ICH) in patients with spontaneous ICH. The current meta-analysis was conducted following Preferred Reporting Items for Systematic Reviews and Meta-analyses (PRISMA) guidelines. A systematic search was performed using PubMed, EMBASE, and Cochrane Library to identify studies assessing the use of statins in patients with ICH. The primary outcome assessed in the current meta-analysis was a hemorrhagic stroke. The secondary outcomes included cardiac-related events and all-cause mortality. A total of 9 studies were included in the current meta-analysis enrolling 49027 patients, with 8094 patients on statin therapy and 40933 patients in the control group. The risk of recurrent ICH was significantly lower in patients receiving stains (RR: 0.81, 95% CI: 0.67-0.99, p-value: 0.02) compared to placebo. However, no significant differences were observed regarding all-cause mortality (RR: 0.80, 95% CI: 0.53-1.20, p-value: 0.27) and cardiovascular events (RR: 1.24, 95% CI: 0.88-1.74). In ICH patients, statins can reduce the risk of recurrent ICH in patients with a history of ICH. However, statins had no significant effect on all-cause mortality and cardiovascular events.

## Introduction and background

Intracerebral hemorrhage (ICH) is the second most common cause of stroke, followed by an ischemic stroke. Nearly 15% of all acute strokes are caused by ICH, which has a high case fatality rate [1]. The ICH rate is expected to double in the next 50 years because of the increasing aging population [2]. Initial treatment goals include preventing the extension of hemorrhage and managing and preventing secondary brain injury and other medical and neurological complications [3]. Despite significant advancements in neurocritical care, more than 50% of ICH patients are at risk of dying or suffering from severe complications [3]. However, the most efficient treatment for ICH remains contentious. Previous reports have shown that 1.3% to 7.4% of ICH patients experience recurrence within one year [4], and 18.8% of patients experience recurrence within five years [5].

 The best way to treat ICH is to limit any further neurologic damage, which includes controlling hematoma growth and taking care of any neurologic issues as soon as they arise. The function of surgical and medical treatment is still up for debate [6]. The penumbra tissue around the hematoma, which is functionally compromised but still possibly alive, is the subject of most of this debate. Depending on the site and extent of the hemorrhage, conservative medical care sometimes takes precedence over surgery [6]. For coagulopathy, factor replacement therapy, vitamin K or platelets are the best medical treatments.

Statins can play an important role in the tertiary and primary prevention of cardiovascular events by reducing cholesterol in stroke patients [7]. One randomized control trial (RCT) has demonstrated that statin withdrawal is linked with poor functional outcomes and increased risk of death [8]. Experimental data has shown that anti‐inflammatory effects, neurodegenerative, neuroprotective, vasodilation, and anti-inflammatory effects can be beneficial in ICH [9]. These beneficial effects can be vital in enhancing clinical outcomes [10].

Two meta-analyses, including RCTs, found no significant relation between statin use and recurrent intracerebral hemorrhage. However, these meta-analyses reported a significant decrease in all-cause mortality and all-stroke types [11-12]. Several retrospective studies showed promising mortality and functional outcomes with an early continuation of statin therapy and poor outcomes in post-ICH patients who discontinued statins [13-14]. According to several meta-analyses, statin use in ICH patients is linked to better functional outcomes and reduced mortality, which suggests that patients who start taking statins or continue taking them after admission are more likely to have positive clinical results [15-16].

Currently, the present recommendations for statin management in patients with ICH are unclear, and the evidence of its risks or benefits is insufficient. American Stroke Association and American Heart Association guidelines for ICH management recommended that insufficient data are there to recommend restrictions on the utilization of statins in patients with ICH that do not change from the past guidelines [17].

Additional analysis is required to provide the highest recommendations or guidelines on statin utilization in ICH patients. The current meta-analysis will provide the most up-to-date and comprehensive evidence for managing patients with ICH, aiding in updating recommendations, putting light on future studies' direction, and guiding the selection of clinical strategies for healthcare professionals worldwide. This meta-analysis aims to assess statin therapy's effect on all-cause mortality and recurrence of ICH in patients with spontaneous ICH.

## Review

Methodology

The current meta-analysis was conducted following Preferred Reporting Items for Systematic Reviews and Meta-analyses (PRISMA) guidelines.

Search strategy

A systematic search was performed using PubMed, EMBASE, and Cochrane Library to identify studies assessing the impacts of statins in patients with spontaneous ICH. The key terms used to search for relevant articles included: "statins", "intracerebral hemorrhage", "mortality," and "outcomes". The search was limited to RCTs, retrospective cohort and prospective cohort studies up to September 2022. Two investigators searched published studies independently, screened the abstracts and titles, and then identified potential studies as per the prespecified inclusion criteria to be included in the current meta-analysis. Eligibility issues were resolved via discussion. A search was also repeated to make sure completeness and accuracy.

Eligibility Criteria

We included all studies, including RCTs and prospective and retrospective cohorts, that treated individuals with ICH, including initiating, restarting, or discontinuing statins after ICH onset. Studies were included in the current meta-analysis if the patients' age was 18 years or more with a history of ICH. The intervention group included patients who received statin therapy, whereas, in the current group, patients received the standard treatment of a placebo. Studies with fewer than six months of follow-up periods were excluded from the current meta-analysis. Lastly, case-control studies, case reports, case series, editorials, and review articles were also not included in the current meta-analysis. The primary outcome assessed in the current meta-analysis was a recurrence of ICH, and the secondary outcomes included cardiac-related events and all-cause mortality.

Data Extraction

Data were extracted using a prespecified data extraction form developed on Microsoft Excel. Data extracted from the included studies for the meta-analysis included: first author name, year of publication, study design, groups, sample size, follow-up period, and outcomes. Data were extracted by two authors independently, and any disagreement between them was resolved by discussion.

Data Analysis

Data analysis was performed using Review Manager (version 5.4.0). Pooled treatment effects for binary endpoints were compared using risk ratios (RRs) and their 95% confidence intervals (CIs). Summary RRs were computed using Mantel-Haenszel random-effects models. The most cautious strategy is the random-effects model since it accounts for both within- and between-study heterogeneity. Forest plots were also drawn, demonstrating the estimates of each study and pooled estimates. A p-value less than 0.05 was considered significant. Heterogeneity was assessed using I-square statistics. Cochran-Q statistics were used to test heterogeneity, and a p-value less than 0.1 was considered significant.

Results

**Figure 1:**
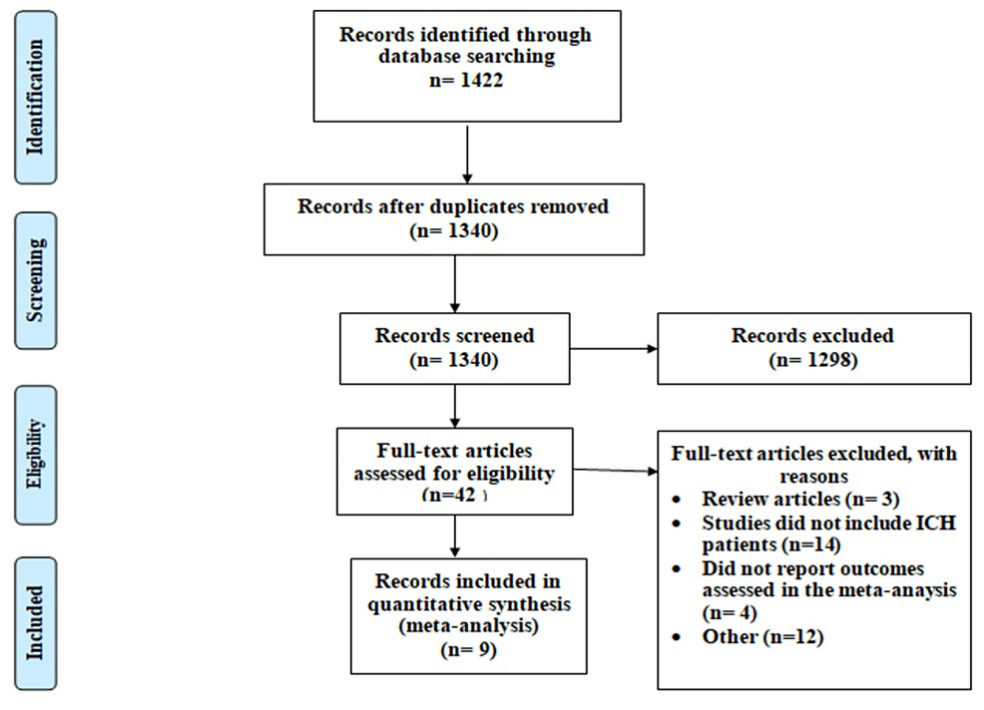
PRISMA flowchart of selection of studies

Figure 1 PRISMA flowchart of screening and selection of studies. We identified 1422 studies, of which 82 records were excluded because of duplicate records. Abstract and title screening was done of 1340 articles, of which 1298 were excluded due to unmet inclusion criteria. Among the 42 articles assessed for eligibility, nine were included in the current meta-analysis [14,18-25].

The study characteristics are shown in Table 1. A total of 49027 patients were included, with 8094 patients on statin therapy and 40933 patients in a control group. Among all included studies, eight were observational [14,18,20-25], while 1 was a post-hoc analysis of RCT [19]. The follow-up period of included studies ranged from 6 months to 120 months.

**Table 1:** Characteristics of the Included Studies

Author Name	Year of Publication	Study Design	Groups	Sample Size	Follow-up Period
Chen et al. [18]	2015	Observational	Statin	749	12 Months
			Control	7583	
Dowlatshahi et al. [14]	2012	Observational	Statin	537	6 Months
			Control	1929	
Goldstein et al. [19]	2007	RCT post-hoc analysis	Statin	45	60 Months
			Control	48	
Lin et al. [20]	2019	Observational	Statin	1338	60 Months
			Control	1338	
Pan et al. [21]	2014	Observational	Statin	220	12 Months
			Control	2998	
Tapia-Perez et al. [22]	2013	Observational	Statin	29	6 Months
			Control	149	
Ribe et al. [23]	2020	Observational	Statin	2728	120 Months
			Control	13640	
Schmidt et al. [24]	2016	Observational	Statin	2258	60 Months
			Control	13012	
Winkler et al. [25]	2013	Observational	Statin	190	12 Months
			Control	236	

Risk of Recurrent ICH

**Figure 2:**
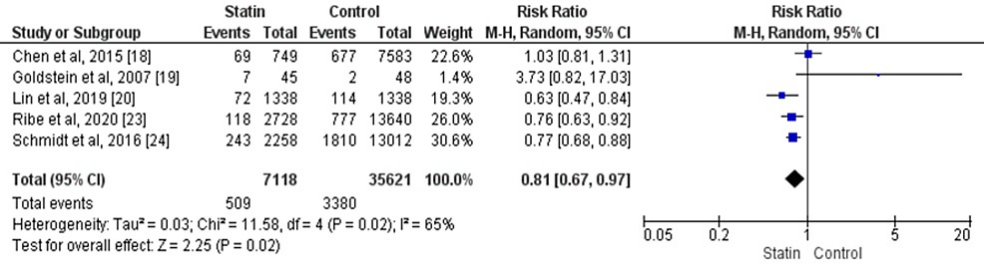
Comparison of Statins versus placebo, Outcome recurrent ICH Sources: References [18-20, 23-24]

A total of five studies reported data on recurrent ICH. The recurrent ICH was observed in 9.09% of patients [18-20,23-24]. Rates were 7.15% and 9.49% for the statin and control groups, respectively. The risk of recurrence of ICH was lower in patients receiving statins than in the placebo group (RR: 0.81, 95% CI: 0.67-0.99, p-value: 0.02). There was significant heterogeneity among the study results (I-square: 65%, p-value: 0.02), as shown in figure 2.

Risk of All-cause Mortality

**Figure 3:**
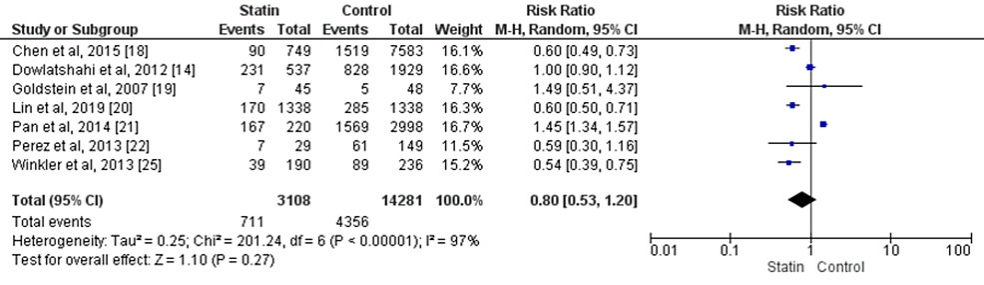
Comparison of Statins versus placebo, Outcome: All-cause Mortality Sources: References [14, 18-22, 25]

Seven studies compared all-cause mortality between ICH patients who received statins and those who did not [14,18-22, 25]. The overall all-cause mortality rate was 29.13%, and no significant difference was found between the two study groups (RR: 0.80, 95% CI: 0.53-1.20, p-value: 0.27). There was significant heterogeneity among the study results (I-square: 97%, p-value: 0.001), as shown in figure 3.

Risk of Cardiovascular Events

**Figure 4:**
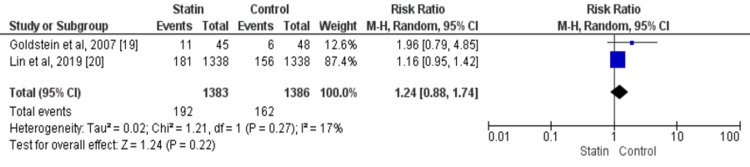
Comparison of Statins versus placebo, Outcome: Cardiovascular events Sources: References [19-20]

In the two studies reporting on cardiovascular events [19-20], the overall rate was 12.78%, without any significant difference between patients who received statin and those who did not (RR: 1.24, 95% CI: 0.88-1.74). No significant heterogeneity was found among the study results (I-square: 17%, p-value: 0.27), as shown in figure 4.

Discussion

In the current meta-analysis, we included nine studies enrolling 42193 patients. We found that statin use in patients with ICH had a beneficial impact in reducing recurrent ICH. For risk of all-cause mortality and cardiovascular events, no significant differences were between the patients who received statins and those who did not.

ICH is a fatal disease with no particular treatment for improving the prognosis. The primary etiology of ICH can be categorized into cerebral amyloid vascular disease (CAA) and hypertension. Secondary risk factors of ICH are brain trauma, coagulation abnormalities, arteriovenous malformation, aneurysm, brain tumors, etc. [26]. In clinical practice, statins are frequently used to lower cholesterol and low-density lipoproteins levels and are frequently used in both primary and secondary prevention of cerebrovascular and cardiovascular illnesses caused by atherosclerosis [27]. Statins decrease plasma cholesterol levels, enhance the permeability of the blood-brain barrier, and inhibit aggregation of platelets, thrombosis, and thrombin-linked reactions after acute ICH, ensuing in further enlargement of cerebral hematoma and poor prognosis [28].

The anticoagulant and antiplatelet impacts of statins have raised questions that they may enhance the hazard of ICH. Statins have also been speculated to have potentially negative effects in acute ICH, where their varied pharmacological characteristics may help to increase hemorrhage [29]. In two randomized control trials that enrolled patients with a previous stroke, it was found that the risk of recurrent ICH was enhanced in patients receiving statins compared with placebo [7, 30]. Of the two RCTs, SPARCL conducted a subgroup analysis of patients with a previous ICH, which showed a non-significant impact of statins on the risk of recurrent ICH [19]. In contrast, our meta-analysis showed that statins could significantly reduce the risk of recurrent ICH among patients with previous ICH. The meta-analysis by Ziff et al. included three studies assessing statins' impact on recurrent ICU [31]. The study found no significant effect of statin on recurrent ICH. However, the study included 12 studies that assessed the effect of statins on all-cause mortality and found that the use of statins was significantly associated with decreased risk of all-cause mortality [31]. We included studies with at least six months of follow-up in the current meta-analysis. Most of the studies in the meta-analysis conducted by Ziff et al. included only observational studies and our meta-analysis also included one posthoc analysis of RCT [19]. In our meta-analysis, we included two studies conducted by Lin et al. [20] and Ribe et al. [23]. Both of these studies favored statins to reduce the risk of ICH in patients with a history of ICH. A study conducted by Lin et al. included patients with dyslipidemia. However, we can reassure the findings because of the observational of the studies. These findings do not support stopping statins after ICH, but large randomized trials are required to consolidate these results.

Predictably, among physicians, stroke utilization in patients after ICH remains argumentative. American guidelines recommended the use of statins in ICH patients because of insufficient data for advising restriction (class IIb; level C). On the other hand, European recommendations do not address this issue [6]. The only double-blinded study that exclusively enrolled patients with ICH was terminated because of poor recruitment (NCT00718328).

The current meta-analysis has certain limitations: The sample is deficient for both observational studies and RCT for generating adequately powered pooled effects, particularly in the ICH cohort; More data was needed to analyze the type of statin and statin; High heterogeneity was reported in the study outcomes because of differences in study designs, follow-up periods, and population type; Due to the non-availability of individual-level data, we could not do a sub-group analysis. To form future guidelines, more RCTs need to be carried out to understand the impact of statins on patients with ICH. 

## Conclusions

The current meta-analysis was conducted to discuss the effect of statins on the risk of ICH in patients with a history of ICH. The current meta-analysis found that statins can reduce the risk of recurrent ICH in patients with a history of spontaneous ICH. However, the current study did not find any significant effect of statin on all-cause mortality and cardiovascular events. The findings of the current meta-analysis were based on observational studies with only one post hoc analysis of RCT. Given that observational studies are subjected to biasness, future randomized control trials are required in patients with ICH survivors to clarify the impact of statins on the future risk of ICH.
